# Early Clinical Experience with Trifluridine/Tipiracil for Refractory Metastatic Colorectal Cancer: The ROS Study

**DOI:** 10.3390/cancers13184514

**Published:** 2021-09-08

**Authors:** Pilar García-Alfonso, Andrés Muñoz, Jerónimo Jiménez-Castro, Paula Jiménez-Fonseca, Carles Pericay, Federico Longo-Muñoz, Carmen Reyna-Fortes, Guillem Argilés-Martínez, Beatriz González-Astorga, María José Gómez-Reina, Ana Ruiz-Casado, Nuria Rodríguez-Salas, Rafael López-López, Alberto Carmona-Bayonas, Verónica Conde-Herrero, Enrique Aranda, on behalf of the ROS Study Group

**Affiliations:** 1 Department of Medical Oncology, Hospital General Universitario Gregorio Marañón, 28007 Madrid, Spain; andresmunmar@hotmail.com; 2 Department of Medical Oncology, Hospital Universitario Virgen del Rocío, 41013 Seville, Spain; jerojc@gmail.com; 3 Department of Medical Oncology, Hospital Universitario Central de Asturias, ISPA, 33011 Oviedo, Spain; palucaji@hotmail.com; 4 Department of Medical Oncology, Hospital Universitari Parc Taulí, 08208 Sabadell, Spain; cpericay@gmail.com; 5 Department of Medical Oncology, Hospital Universitario Ramón y Cajal, 28034 Madrid, Spain; fedelongomunoz@hotmail.com; 6 Department of Medical Oncology, UGC Intercentros de Oncología Médica, Hospitales Universitarios Regional y Virgen de la Victoria, IBIMA, 29010 Málaga, Spain; c.reyna.fortes@gmail.com; 7 Department of Medical Oncology, Hospital Universitari Vall d’Hebrón, 08035 Barcelona, Spain; gargiles@vhio.net; 8 Department of Medical Oncology, Hospital Universitario Clínico San Cecilio, 18016 Granada, Spain; bea_astorga@hotmail.com; 9 Department of Medical Oncology, Hospital Universitario Puerta del Mar, 11009 Cádiz, Spain; mariajose.gomezreina@gmail.com; 10 Department of Medical Oncology, Hospital Universitario Puerta de Hierro Majadahonda, 28222 Majadahonda, Spain; arcasado@salud.madrid.org; 11 Department of Medical Oncology, Hospital Universitario La Paz, CIBERONC, 28046 Madrid, Spain; nuria.rodriguez@salud.madrid.org; 12 Translational Medical Oncology Group, Department of Medical Oncology, Hospital Clínico Universitario e Instituto de Investigación Sanitaria (IDIS), CIBERONC, Facultad de Medicina de la Universidad de Santiago de Compostela, 15706 Santiago de Compostela, Spain; rafael.lopez.lopez@sergas.es; 13 Department of Medical Oncology, Hospital General Universitario Morales Meseguer, 30008 Murcia, Spain; alberto.carmonabayonas@gmail.com; 14 Department of Medical Oncology, Hospital Universitario Virgen de las Nieves, 18014 Granada, Spain; berenice2es@yahoo.es; 15 Department of Medical Oncology, Hospital Universitario Reina Sofía, IMIBIC, Universidad de Córdoba, CIBERONC, Instituto de Salud Carlos III, 14004 Córdoba, Spain; earandaa@seom.org

**Keywords:** biomarkers, chemotherapy, colorectal cancer, combination therapy, real-life, trifluridine/tipiracil

## Abstract

**Simple Summary:**

Trifluridine/tipiracil is an oral combination therapy currently approved as a salvage-line treatment in patients with metastatic colorectal cancer refractory to, or not, candidates for available therapies. However, there is no consensus on the specific factors that should be considered to select patients who benefit the most from trifluridine/tipiracil in clinical practice. The aim of our retrospective cohort study was to assess the early clinical experience with trifluridine/tipiracil in Spain and identify potential survival markers. Our findings endorse the real-life efficacy and safety of trifluridine/tipiracil for refractory metastatic colorectal cancer, as well as revealing the presence of ≤2 metastatic sites, absence of liver metastasis, alkaline phosphatase levels < 300 IU, trifluridine/tipiracil dose reductions, and neutrophil/lymphocyte ratio < 5 as survival markers. Combinations of these markers may help physicians to identify subsets of patients with refractory metastatic colorectal cancer that may benefit the most from trifluridine/tipiracil in their daily practice.

**Abstract:**

Trifluridine/tipiracil is currently approved for metastatic colorectal cancer (mCRC) refractory to available therapies. However, there is no consensus on factors that predict treatment outcomes in daily practice. We assessed the early clinical experience with trifluridine/tipiracil in Spain and potential survival markers. This was a retrospective cohort study of mCRC patients who participated in the trifluridine/tipiracil early clinical experience programme in Spain. The primary outcome was overall survival (OS). Associations between OS and patient characteristics were assessed using multivariate Cox regression analyses. A total of 379 patients were included in the study. Trifluridine/tipiracil was administered for a median of 3.0 cycles and discontinued mainly due to disease progression (79.2%). The median OS was 7.9 months, with a 12-month OS rate of 30.5%. Cox analyses revealed that the following variables independently enhanced OS: ≤2 metastatic sites, no liver metastasis, alkaline phosphatase < 300 IU, trifluridine/tipiracil dose reductions, and neutrophil/lymphocyte ratio < 5. Grade ≥ 3 toxicities were reported in 141 (37.2%) patients, including mainly afebrile neutropaenia (23.2%), anaemia (12.1%), and thrombocytopaenia (5.3%). This study supports the real-life efficacy and safety of trifluridine/tipiracil for refractory mCRC and identifies tumour burden, liver metastasis, alkaline phosphatase, dose reductions, and neutrophil/lymphocyte ratio as survival markers.

## 1. Introduction

Colorectal cancer is one of the three most commonly diagnosed cancers and the second leading cause of cancer death worldwide [1]. Only 39% of colorectal cancer patients are diagnosed with localised disease, and despite the improvements achieved in its management, the 5-year survival of patients with distant metastases drops to 14% [2]. Current therapies for metastatic colorectal cancer (mCRC) involve several active drugs administered either as monotherapy or in combination, including cytotoxic agents such as fluoropyrimidines, irinotecan or oxaliplatin, and targeted therapies against epidermal growth factor receptor (EGFR) or vascular endothelial growth factor (VEGF) [3,4]. However, the therapeutic management of mCRC refractory to these therapies remains challenging.

Trifluridine/tipiracil is an oral combination therapy consisting of an antineoplastic thymidine-based nucleoside analogue (trifluridine) and a thymidine phosphorylase inhibitor (tipiracil hydrochloride). Trifluridine is the active cytotoxic component, responsible for preventing tumour cell proliferation by interfering with DNA function [5,6], while tipiracil improves trifluridine bioavailability by inhibiting its catabolism [7,8]. Trifluridine/tipiracil administration is currently approved as a salvage-line treatment in patients with mCRC refractory to, or not candidates for, available therapies based on the pivotal phase III RECOURSE trial. Its findings revealed a median overall survival (OS) of 7.1 months with trifluridine/tipiracil versus 5.3 months with placebo [9]. Trifluridine/tipiracil improved OS irrespective of age, *KRAS* status, time from first metastasis or geographic region, and also enhanced progression-free survival (PFS), disease control, and performance status [9,10,11]. Subsequent post hoc analyses also pointed to neutropaenia as a surrogate marker of trifluridine/tipiracil efficacy [12], and supported low tumour burden, indolent disease, and absence of liver metastasis as prognostic factors [13].

Compassionate use programmes and cohort studies endorsed the use of trifluridine/tipiracil in non-trial conditions [14,15,16,17,18,19]. Many of them also addressed the unmet need of identifying prognostic or predictive factors, which included pre-treatment performance status [15,16,18,19,20], *KRAS* status [18], time to metastasis [19], number of metastases [15], time from metastasis diagnosis [19] or first-line therapy [17], alkaline phosphatase levels [15,18], platelet and leucocyte counts [15,18], and neutrophil/leucocyte ratio [15,20], as well as on-treatment neutropaenia [15,17,19,21], and dose reductions [15,16]. However, there is no consensus on the specific factors that should be considered to select patients who benefit the most from trifluridine/tipiracil.

In light of the above, this study aimed to provide further insights into the efficacy and safety of trifluridine/tipiracil for refractory mCRC in the early clinical experience in Spain, and to identify factors that may help physicians to predict better outcomes in daily practice.

## 2. Materials and Methods

ROS was a retrospective cohort study conducted in the Departments of Medical Oncology at 35 Spanish hospitals according to Good Pharmacoepidemiology Practices, the Declaration of Helsinki, and national regulations. The study was approved by the ethics committee of Hospital General Universitario Gregorio Marañón (Madrid, Spain) and alive patients gave their written informed consent; informed consent was waived in deceased and lost-to-follow-up patients.

### 2.1. Patient Population

The study included patients aged over 18 years, with histologically or cytologically confirmed colorectal adenocarcinoma, and who had participated in the early clinical experience programme for trifluridine/tipiracil. To participate in this program, patients had to be previously treated with, or not considered candidates for, available therapies (fluoropyrimidine-, oxaliplatin-, or irinotecan-based chemotherapies, anti-VEGF or anti-EGFR agents). Eligible patients must have completed the early clinical treatment with trifluridine/tipiracil at study enrolment. Patients who had received other investigational drugs or anticancer therapies (chemotherapy, immunotherapy, biological response modifiers, or endocrine therapy) while receiving trifluridine/tipiracil were excluded.

### 2.2. Study Treatment

Trifluridine/tipiracil (Lonsurf^®^, Laboratoires Servier, Suresnes, France) was taken orally at a starting dose decided at the discretion of the treating oncologist and administered twice daily, after breakfast and dinner, on days 1 to 5 and 8 to 12 of each 28-day cycle. Patients were treated by the supervising physicians according to the recommendations included in the product information. Dose adjustments, delays, and discontinuations were performed according to clinical criteria.

### 2.3. Assessments

All study data were retrospectively collected from patient medical charts, progress reports submitted to the health authorities, and a data log owned by Laboratorios Servier S.L. containing previously collected information on compassionate use of trifluridine/tipiracil.

These data included demographics and baseline clinical characteristics, including the Eastern Cooperative Oncology Group (ECOG) performance status, medical history of colorectal cancer, and antitumour therapies prior to trifluridine/tipiracil. Dates and doses of trifluridine/tipiracil were collected at the first treatment cycle, along with subsequent treatment modifications and the total number of administered cycles. Data available on follow-up ECOG performance status, radiologic progression and best response to trifluridine/tipiracil according to Response Evaluation Criteria in Solid Tumours (version 1.1) were also retrieved [22], as well as grade ≥ 3 adverse events attributed to trifluridine/tipiracil. These events were coded using the Medical Dictionary for Regulatory Activities (MedDRA) and their severity graded by National Cancer Institute Common Toxicity Criteria (version 4.03) [23]. Other therapies received after ending trifluridine/tipiracil and the survival status (alive, dead, or lost to follow-up) at data collection were also retrieved.

### 2.4. Outcomes

The primary study outcome was OS, which was defined as the time from trifluridine/tipiracil start to patient death from any cause. Secondary efficacy outcomes included PFS measured as the time from trifluridine/tipiracil start to radiologic disease progression or death from any cause, and PFS measured as the time from trifluridine/tipiracil start to clinical disease progression or death from any cause. Other secondary efficacy outcomes were OS and PFS rates at 2, 4, 6, 8, 10, and 12 months, overall response rate (percentage of patients with a complete or partial response), disease control rate (percentage of patients with complete or partial response or stable disease, with stable disease assessed at least 6 weeks after starting trifluridine/tipiracil), changes in ECOG performance status (improved, maintained, or worsened), and associations between OS and patient characteristics. Trifluridine/tipiracil exposure/management and grade ≥ 3 treatment-related adverse events were additional secondary outcomes.

### 2.5. Statistical Considerations

Sample size calculation was based on the median OS reported in the RECOURSE trial [9], a two-sided test, power of 99%, type I error of 0.01, loss rate ≤ 10%, and maximum follow-up of 12 months. The estimated sample size was 467 patients, for which the lower and higher critical values were 6.1 and 8.4 months, respectively.

Kaplan–Meier analyses were performed to assess OS and PFS, including survival curves, median estimates, and 95% confidence intervals (CIs). Descriptive statistics were used to determine survival rates, overall response rate, disease control rate, changes in ECOG performance status, trifluridine/tipiracil exposure/management, and treatment-related adverse events. Bivariate Cox regression analyses assessed associations between OS and the following characteristics: age ≥ 65 or <65 years, sex, ECOG performance status 0–1 or ≥2, time from metastasis diagnosis < 18 or ≥18 months, number of metastatic sites ≤2 or ≥3, presence or absence of liver metastatic lesions, synchronous or metachronous metastases, right or left colon primary tumour location, primary tumour molecular status (*KRAS*, *RAS* [*KRAS* + *NRAS*], *BRAF*, *PI3K*, *HER2*, and microsatellite instability), primary tumour surgery, previous antitumour lines < 3 or ≥3, and laboratory data prior to the first trifluridine/tipiracil cycle (platelet count ≥ 400 × 10^9^/L or <400 × 10^9^/L, leukocyte count ≥ 10 × 10^9^/L or <10 × 10^9^/L, alkaline phosphatase level ≥ 300 or <300 IU, and haemoglobin level ≥ 11 or <11 g/dL). A multivariate Cox regression model was built using characteristics with *p* < 0.20 in bivariate analyses, including the calculation of hazard ratios (HRs) and 95% CIs. Another multivariate Cox regression model was built adding neutropaenia, neutrophil/lymphocyte ratio, and dose reduction to the previously mentioned characteristics. OS in the prognostic subsets of patients with low tumour burden (≤2 metastatic sites), indolent disease (≥18 months from metastasis diagnosis), and absence of liver metastases [13] was also explored using log-rank tests and Cox regressions.

Missing data were not considered in the analyses, and a significance level of 0.05 was used for statistical testing. The statistical analyses were performed with IBM SPSS Statistics version 22.0 (IBM Corp., Armonk, NY, USA).

## 3. Results

### 3.1. Patient Characteristics

A total of 402 patients were screened between June and November 2019, 23 of whom were not eligible (Supplementary Figure S1). Thus, 379 patients were finally included in this study, whose baseline characteristics and tumour molecular status are described in Table 1 and Table 2, respectively.

### 3.2. Study Treatment

The planned dose of trifluridine/tipiracil at the first cycle ranged from 25.0 to 35.0 mg/m^2^ twice daily, with a median (interquartile range, IQR) of 35.0 (35.0–35.0) mg/m^2^ (Supplementary Table S1). A total of 116 patients needed at least one of the 145 dose reductions and 191 patients needed at least one of the 294 dose delays reported during the trifluridine/tipiracil treatment. The median (IQR) number of administered cycles was 3.0 (2.0–4.0), and end of treatment was due mainly to disease progression (79.2%), followed by general state impairment (12.7%), toxicity (4.5%), patient decision (1.8%), and other reasons (1.8%).

One hundred and fifty-five (40.9%) patients received other anticancer therapies after trifluridine/tipiracil, including: chemotherapy *n* = 78 (20.6%; capecitabine *n* = 24, 5-fluorouracil *n* = 46, oxaliplatin *n* = 33, and irinotecan *n* = 23), anti-EGFR therapies *n* = 15 (4.0%; cetuximab *n* = 7, and panitumumab *n* = 8), anti-VEGF therapies *n* = 32 (8.4%; bevacizumab *n* = 30, and aflibercept *n* = 2), regorafenib *n* = 51 (13.5%), and other therapies *n* = 39 (10.3%).

### 3.3. Efficacy

**Figure 1:**
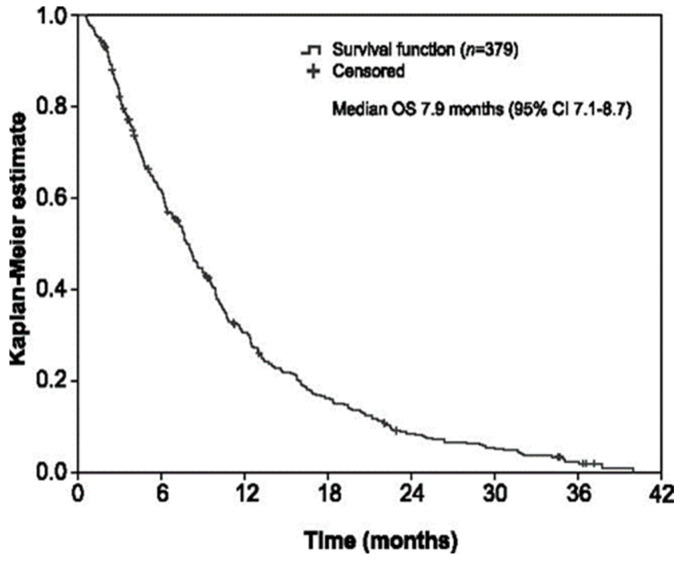
Kaplan–Meier plot for overall survival. CI: confidence interval, OS: overall survival.

**Figure 2:**
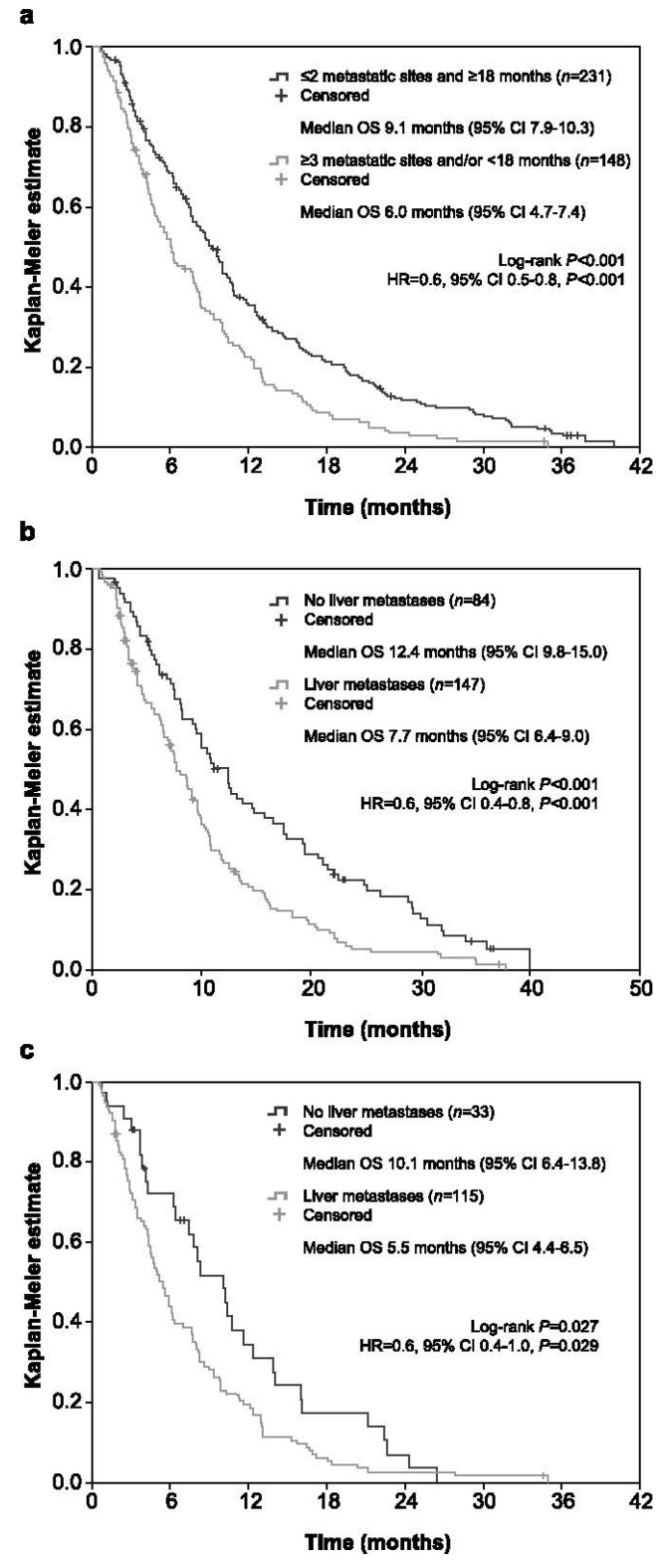
Kaplan–Meier plots for overall survival in the exploratory analysis of prognostic subsets. They included the comparison of patients with ≤2 metastatic sites and ≥18 months from metastasis diagnosis versus those with ≥3 metastatic sites and/or <18 months (**a**), patients with ≤2 metastatic sites, ≥18 months from metastasis diagnosis and absence of liver metastasis versus those with ≤2 metastatic sites, ≥18 months from metastasis diagnosis and liver metastasis (**b**), and patients with ≥3 metastatic sites and/or <18 months from metastasis diagnosis and absence of liver metastasis versus those with ≥3 metastatic sites and/or <18 months from metastasis diagnosis and liver metastasis (**c**). CI: confidence interval, HR: hazard ratio, OS: overall survival.

After a median (IQR) follow-up of 7.6 (3.7–12.9) months from the start of trifluridine/tipiracil, the median OS was 7.9 months (95% CI 7.1–8.7; Figure 1). The OS rates at 2, 4, 6, 8, 10, and 12 months were 92.6% (95% CI 90.0–95.2%), 74.5% (95% CI 70.1–78.9%), 61.5% (95% CI 56.5–66.4%), 49.1% (95% CI 44.0–54.2%), 37.7% (95% CI 32.8–42.7%), and 30.5% (95% CI 25.7–35.2%), respectively. The multivariate Cox regression analysis revealed longer OS in patients with ≤2 metastatic sites (HR = 0.6, 95% CI 0.5–0.8, *p* < 0.001), absence of liver metastasis (HR = 0.7, 95% CI 0.5–0.9, *p* = 0.004), and alkaline phosphatase levels < 300 IU (HR = 0.6, 95% CI 0.4–0.8, *p* < 0.001) (Table 3). When neutropaenia, neutrophil/lymphocyte ratio, and dose reduction were included in the multivariate Cox regression analysis, longer OS was associated with ≤2 metastatic sites (HR = 0.6, 95% CI 0.5–0.8, *p* < 0.001), alkaline phosphatase levels <300 IU (HR = 0.5, 95% CI 0.4–0.7, *p* < 0.001), dose reductions (HR = 0.6, 95% CI 0.4–0.8, *p* < 0.001), and neutrophil/lymphocyte ratio < 5 (HR = 0.5, 95% CI 0.4–0.7, *p* < 0.001) (Table 3). Furthermore, the exploratory analysis of prognostic subsets with low tumour burden and indolent disease revealed longer OS in patients with ≤2 metastatic sites and ≥18 months from metastasis diagnosis than those with ≥3 metastatic sites and/or <18 months (HR = 0.6, 95% CI 0.5–0.8, *p* < 0.001; Figure 2a). The OS was even longer when liver metastases were absent versus present in both subsets of patients: ≤2 metastatic sites and ≥18 months (HR = 0.6, 95% CI 0.4–0.8, *p* < 0.001; Figure 2b) and ≥3 metastatic sites and/or <18 months (HR = 0.6, 95% CI 0.4–1.0, *p* = 0.029; Figure 2c).

**Figure 3:**
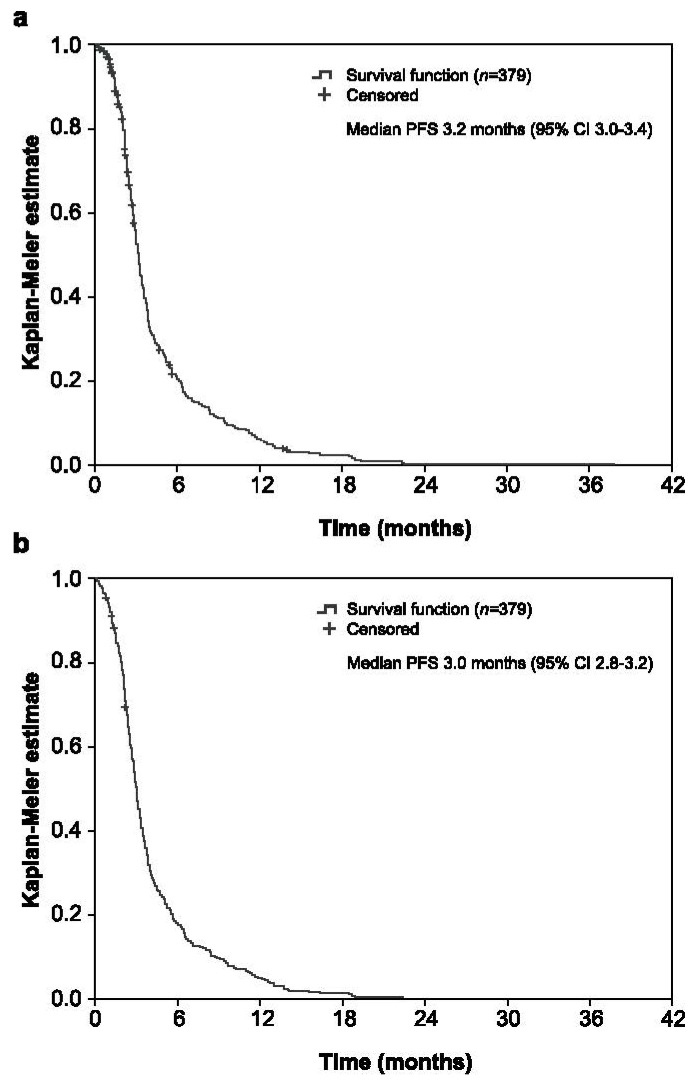
Kaplan–Meier plots for progression-free survival considering radiologic disease progression (**a**) and clinical disease progression (**b**). CI: confidence interval, PFS: progression-free survival.

The median PFS measured until radiologic disease progression or death was 3.2 months (95% CI 3.0–3.4; Figure 3a), with PFS rates at 2, 4, 6, 8, 10, and 12 months of 82.9% (95% CI 79.0–86.8%), 34.1% (95% CI 29.1–39.2%), 21.3% (95% CI 16.9–25.6%), 14.5% (95% CI 10.7–18.3%), 9.8% (95% CI 6.6–13.1%), and 6.4% (95% CI 3.7–9.0%), respectively. When its definition included clinical disease progression, the median PFS was 3.0 months (95% CI 2.8–3.2; Figure 3b) and PFS rates at 2, 4, 6, 8, 10, and 12 months were 77.2% (95% CI 72.9–81.4%), 30.7% (95% CI 26.1–35.4%), 18.4% (95% CI 14.5–22.4%), 12.6% (95% CI 9.2–15.9%), 8.3% (95% CI 5.5–11.1%), and 5.3% (95% CI 3.1–7.6%), respectively.

ECOG performance status was assessed in 375 patients during trifluridine/tipiracil treatment, which was maintained in 176 (46.9%) patients and improved in 31 (8.3%). Worsening of ECOG performance status was observed in 168 (44.8%) patients.

### 3.4. Safety

A total of 141 (37.2%) patients reported having experienced at least one grade ≥3 treatment-related adverse event, which mainly included (frequency ≥ 1%): afebrile neutropaenia (23.2%), anaemia (12.1%), thrombocytopaenia (5.3%), diarrhoea (4.0%), asthenia (3.4%), febrile neutropaenia (2.9%), nausea (2.1%), and vomiting (1.3%) (Supplementary Table S2).

## 4. Discussion

The results from the ROS cohort study support the efficacy and safety of trifluridine/tipiracil for refractory mCRC in the early clinical experience in Spain. More than half of patients maintained or even improved their performance status, nearly one-third achieved disease control, and median PFS and OS reached 3 months and almost 8 months, respectively. These results are in line with the benefits observed with trifluridine/tipiracil in the RECOURSE trial [9,10,11], along with other compassionate use programmes and observational studies that confirm the use of trifluridine/tipiracil as a feasible treatment alternative for refractory mCRC in a real-life setting [14,15,18,19,20].

PFS is a commonly used outcome for refractory mCRC that is usually reported until radiologic disease progression or death. However, the real disease progression usually occurs between two radiologic assessments [14,24], and the data available on PFS measured until clinical progression or death is still limited. This study shed some light on this issue, supporting the achievement of similar findings when considering clinical progression. Nonetheless, it is noteworthy that PFS seems to be poorly correlated with OS after second-line treatment and OS remains the most robust outcome estimate when assessing treatment efficacy for mCRC [25]. In this regard, our findings also revealed that the median OS might even reach 12.4 months in the subset of patients with ≤2 metastatic sites, ≥18 months from metastasis diagnosis, and absence of liver metastasis. These data support the exploratory analysis of Tabernero et al. [13], who defined a subgroup of patients with good prognostic characteristics including low tumour burden (<3 metastatic sites) and indolent disease (≥18 months from metastasis) and another with poor prognostic characteristics that included high tumour burden and/or aggressive disease. The reported median OS in these subgroups were 9.3 and 5.3 months, respectively. Our findings are in line with these data, showing that the 231 patients with ≤2 metastatic sites and ≥18 months from metastasis showed a median OS of 9.1 months versus 6.0 months in the 148 patients with ≥3 metastatic sites and/or <18 months. However, as in our study, the best prognosis could be identified within the subset of patients with good prognostic factors and absence of liver metastasis, with a median OS that reached 16.4 months [13].

In addition to the tumour burden and liver metastasis, our study supports the role of alkaline phosphatase levels, dose reductions, and neutrophil/lymphocyte ratios as survival markers. These findings agree with recently published observational studies that also revealed longer OS in patients with a single metastatic site [15], liver metastases [26], alkaline phosphatase levels < 200 or ≤500 IU/L [15,18], trifluridine/tipiracil dose reductions [15,16], and neutrophil/lymphocyte ratios < 5 [15,20]. However, they also found the potential influence of other factors such as ECOG performance status [15,16,18,19,20,26], platelet count ≤ 350 × 10^9^/L [15], *KRAS* status [18], time to synchronous or metachronous metastasis [19], leukocyte count < 8 × 10^9^/L [18], or neutropaenia as an adverse event [15,17,19,21], which did not independently affect survival in our study. Based on these findings, some attempts have been made to achieve nomograms to screen for the patients who can benefit the most from trifluridine/tipiracil. These include the nomogram reported by Fernández-Montes et al. (2020) in mCRC patients treated with trifluridine/tipiracil, which considered the ECOG performance status, presence of multiple metastatic sites, carcinoembryonic antigen > 10 ng/mL, platelet counts > 350 × 10^9^/L, and phosphatase > 500 IU/L [15]. Another was the Colon Life nomogram, based on refractory mCRC patients treated with trifluridine/tipiracil, regorafenib, or other treatments, that included the ECOG performance status, primary tumour resection, lactate dehydrogenase, and peritoneal involvement [27]. Likewise, the REGOTAS study developed a scoring system to predict OS after trifluridine/tipiracil, including the ECOG performance status, aspartate transaminase > 40 IU/L, C-reactive protein ≥ 1.0 mg/dL, and cancer antigen 19–9 > 37.0 U/mL, as well as another for regorafenib including aspartate transaminase > 40 IU/L, C-reactive protein ≥ 1.0 mg/dL, number of metastatic sites ≥ 3, and <18 months from first-line chemotherapy [26]. A common scoring model for trifluridine/tipiracil and regorafenib was also reported based on the ECOG performance status, ≤18 months form metastasis diagnosis, and prior chemotherapy for ≥2 months beyond the progressive disease [28]. Furthermore, the REBECCA study proposed another score for regorafenib based on the ECOG performance status, time from metastasis diagnosis, initial regorafenib dose, number of metastatic sites, liver metastases, and *KRAS* mutations [29]. However, no specific nomogram or scoring system has been validated for refractory mCRC, and further assessments of prognostic/predictive factors are still needed to make the best therapeutic decisions.

The safety profile of trifluridine/tipiracil was consistent with that previously reported in clinical trials [30], compassionate use programmes [17,18,19], and other observational studies [15,16,20,21]. No new safety concern arose and the main ≥3 grade treatment-related adverse events included afebrile neutropaenia, anaemia, and thrombocytopaenia. In addition, most of these adverse events could be managed with dose reductions or delays, and only led to treatment discontinuation in 4.5% of patients.

Although the prescribing conditions of compassionate use programmes are less strict than clinical trials and more clearly reflect routine clinical practice, the retrospective data extraction from medical charts entailed limitations due to data availability. We cannot therefore exclude the potential underestimation of treatment-related adverse events, or the influence of missing data in the lack of association observed between tumour molecular markers and OS. Likewise, we cannot discern how the low prevalence of variables such as the mutated *KRAS* might have affected the study findings, and the absence of association between ECOG performance status and OS is likely derived from the limited number of patients with ECOG 2. Another limitation is the absence of a central review of scans performed at each participating site. Furthermore, the study design included no comparator group, which would have enabled the magnitude of improvement derived from trifluridine/tipiracil to be quantified. Likewise, a comparator group would have been needed to assess whether the identified survival markers might also affect patients not receiving trifluridine/tipiracil and further clarify their role in colorectal cancer. However, it is noteworthy that this study assessed a wide range of patient characteristics as potential markers of survival and provides additional insight into real-life use of trifluridine/tipiracil in 35 Spanish hospitals, enhancing the generalisability of its findings and expanding the information to consider when treating mCRC in daily practice.

## 5. Conclusions

This study endorses the real-life efficacy and safety of trifluridine/tipiracil for refractory mCRC, as well as supporting the role of specific survival markers such as tumour burden, liver metastasis, alkaline phosphatase, dose reductions, and neutrophil/lymphocyte ratio. Combinations of these markers may help physicians to identify subsets of patients that may benefit the most from trifluridine/tipiracil. Further studies are still needed to confirm our findings, support the most appropriate combination of prognostic/predictive factors to optimise trifluridine/tipiracil treatment, and clarify the role of the best supportive care in patients supposed to have poor response to chemotherapy and worse survival.

## Figures and Tables

**Table 1:** Baseline patient characteristics (*n* = 379).

Characteristics	Value
Age (years)	
Median (IQR)	65 (58–71)
≥65 years, *n* (%)	189 (49.9)
≥70 years, *n* (%)	108 (28.5)
Male, *n* (%)	226 (59.6)
Caucasian, *n* (%)	355 (93.7)
ECOG performance status, *n* (%)	
0	117 (30.9)
1	255 (67.3)
2	7 (1.8)
Site of primary tumour, *n* (%)	
Colon	222 (58.6)
Rectum	129 (34.0)
Colon and rectum	27 (7.1)
Unknown	1 (0.3)
Primary tumour surgery, *n* (%)	316 (83.4)
Timing of metastases from initial diagnosis, *n* (%)	
Synchronous (≤6 months)	231 (60.9)
Metachronous (>6 months)	148 (39.1)
Site of metastasis (frequency ≥10%), *n* (%) ^a^	
Liver	262 (69.1)
Lung	256 (67.5)
Peritoneum	93 (24.5)
Distant node	70 (18.5)
Bone	29 (7.7)
Number of metastatic sites, *n* (%)	
≤2	279 (73.6)
≥3	100 (26.4)
Lines of treatment, *n* (%)	
<3	126 (33.2)
≥3	253 (66.8)
Previous therapies in ≥1 line, *n* (%) ^a^	
Fluoropyrimidines	379 (100)
Oxaliplatin	333 (87.9)
Irinotecan	378 (99.7)
Anti-VEGF	339 (89.4)
Anti-EGFR	117 (46.7)
Regorafenib	60 (15.8)
Other	83 (21.9)
Time from the initial diagnosis to start trifluridine/tipiracil (years), median (IQR)	3.3 (2.2–5.3)
Time from metastasis diagnosis to start trifluridine/tipiracil (years)	
Median (IQR)	2.5 (1.7–4.2)
≥18 months, *n* (%)	320 (84.4)

ECOG: Eastern Cooperative Oncology Group, EGFR: epidermal growth factor receptor, IQR: interquartile range, VEGF: vascular endothelial growth factor. ^a^ Multi-response variable.

**Table 2:** Primary tumour molecular status (*n* = 379).

Molecular Status	Value
Microsatellite instability, *n* (%)	
No	128 (33.8)
Yes	50 (13.2)
Unknown	201 (53.0)
*KRAS* status, *n* (%)	
Wild type	179 (47.2)
Mutated	175 (46.2)
Unknown	25 (6.6)
*RAS (KRAS + NRAS)* status, *n* (%)	
Wild type	88 (23.2)
Mutated	190 (50.1)
Unknown	101 (26.6)
*BRAF* status, *n* (%)	
Wild type	71 (18.7)
Mutated	7 (1.8)
Unknown	301 (79.4)
*PI3K* status, *n* (%)	
Wild type	24 (6.3)
Mutated	6 (1.6)
Unknown	349 (92.1)
*HER2* status, *n* (%)	
Positive	2 (0.5)
Negative	22 (5.8)
Unknown	355 (93.7)

**Table 3:** Patient characteristics associated with overall survival in multivariate Cox analyses.

Characteristics	OS (Months)	Cox 1		Cox 2	
	Median (95% CI)	HR (95% CI)	*p*	HR (95% CI)	*p*
Number of metastatic sites					
≤2	8.6 (7.5–9.7)	0.6 (0.5–0.8)	<0.001	0.6 (0.5–0.8)	<0.001
≥3 ^a^	5.6 (4.7–6.6)				
Liver metastasis					
No	10.7 (8.8–12.7)	0.7 (0.5–0.9)	0.004	-	-
Yes ^a^	6.6 (5.6–7.5)				
Alkaline phosphatase level					
<300 IU	9.8 (8.5–11.0)	0.6 (0.4–0.8)	<0.001	0.5 (0.4–0.7)	<0.001
≥300 IU ^a^	4.1 (2.5–5.7)				
Dose reductions					
Without dose reductions ^a^	6.4 (5.1–7.6)	-	-	0.6 (0.4–0.8)	<0.001
With dose reductions	10.7 (8.8–12.7)				
Neutrophil/lymphocyte ratio					
<5	9.0 (8.0–10.0)	-	-	0.5 (0.4–0.7)	<0.001
≥5 ^a^	4.1 (3.0–5.1)				

CI: confidence interval, HR: hazard ratio, OS: overall survival. ^a^ Reference category.

## Data Availability

All data relevant to the study are included in the article or uploaded as supplementary information. Further data are available from the authors upon reasonable request and with permission of the Spanish Cooperative Group for the Treatment of Digestive Tumours (TTD).

## Supplementary Materials

The following are available online at https://www.mdpi.com/article/10.3390/cancers13184514/s1, Supplementary Figure S1: Summary of patient disposition, Supplementary Table S1: Trifluridine/tipiracil exposure and management (*n* = 379), Supplementary Table S2: Grade ≥ 3 treatment-related adverse events (*n* = 379).

## References

[1] Bray F., Ferlay J., Soerjomataram I., Siegel R.L., Torre L.A., Jemal A. (2018). Global cancer statistics 2018: GLOBOCAN estimates of incidence and mortality worldwide for 36 cancers in 185 countries. CA Cancer J. Clin..

[2] American Cancer Society (2017). Colorectal Cancer Facts & Figures 2017–2019.

[3] National Comprehensive Cancer Network NCCN Clinical Practice Guidelines in Oncology (NCCN Guidelines). Colon Cancer. Version 4.2020. https://www.nccn.org/professionals/physician_gls/pdf/colon.pdf.

[4] Van Cutsem E., Cervantes A., Adam R., Sobrero A., van Krieken J.H., Aderka D., Aranda Aguilar E., Bardelli A., Benson A., Bodoky G. (2016). ESMO consensus guidelines for the management of patients with metastatic colorectal cancer. Ann. Oncol..

[5] Emura T., Suzuki N., Yamaguchi M., Ohshimo H., Fukushima M. (2004). A novel combination antimetabolite, TAS-102, exhibits antitumor activity in FU-resistant human cancer cells through a mechanism involving FTD incorporation in DNA. Int. J. Oncol..

[6] Tanaka N., Sakamoto K., Okabe H., Fujioka A., Yamamura K., Nakagawa F., Nagase H., Yokogawa T., Oguchi K., Ishida K. (2014). Repeated oral dosing of TAS-102 confers high trifluridine incorporation into DNA and sustained antitumor activity in mouse models. Oncol. Rep..

[7] Fukushima M., Suzuki N., Emura T., Yano S., Kazuno H., Tada Y., Yamada Y., Asao T. (2000). Structure and activity of specific inhibitors of thymidine phosphorylase to potentiate the function of antitumor 2′-deoxyribonucleosides. Biochem. Pharmacol..

[8] Emura T., Suzuki N., Fujioka A., Ohshimo H., Fukushima M. (2005). Potentiation of the antitumor activity of α, α, α-trifluorothymidine by the co-administration of an inhibitor of thymidine phosphorylase at a suitable molar ratio in vivo. Int. J. Oncol..

[9] Mayer R.J., Van Cutsem E., Falcone A., Yoshino T., Garcia-Carbonero R., Mizunuma N., Yamazaki K., Shimada Y., Tabernero J., Komatsu Y. (2015). Randomized trial of TAS-102 for refractory metastatic colorectal cancer. N. Engl. J. Med..

[10] Longo-Muñoz F., Argiles G., Tabernero J., Cervantes A., Gravalos C., Pericay C., Gil-Calle S., Mizuguchi H., Carrato-Mena A., Limón M.L. (2017). Efficacy of trifluridine and tipiracil (TAS-102) versus placebo, with supportive care, in a randomized, controlled trial of patients with metastatic colorectal cancer from Spain: Results of a subgroup analysis of the phase 3 RECOURSE trial. Clin. Transl. Oncol..

[11] Van Cutsem E., Mayer R.J., Laurent S., Winkler R., Grávalos C., Benavides M., Longo-Munoz F., Portales F., Ciardiello F., Siena S. (2018). The subgroups of the phase III RECOURSE trial of trifluridine/tipiracil (TAS-102) versus placebo with best supportive care in patients with metastatic colorectal cancer. Eur. J. Cancer.

[12] Yoshino T., Cleary J., Van Cutsem E., Mayer R., Ohtsu A., Shinozaki E., Falcone A., Yamazaki K., Nishina T., Garcia-Carbonero R. (2020). Neutropenia and survival outcomes in metastatic colorectal cancer patients treated with trifluridine/tipiracil in the RECOURSE and J003 trials. Ann. Oncol..

[13] Tabernero J., Argiles G., Sobrero A.F., Borg C., Ohtsu A., Mayer R.J., Vidot L., Moreno Vera S.R., Van Cutsem E. (2020). Effect of trifluridine/tipiracil in patients treated in RECOURSE by prognostic factors at baseline: An exploratory analysis. ESMO Open.

[14] Andersen S.E., Andersen I.B., Jensen B.V., Pfeiffer P., Ota T., Larsen J.S. (2019). A systematic review of observational studies of trifluridine/tipiracil (TAS-102) for metastatic colorectal cancer. Acta Oncol..

[15] Fernandez Montes A., Vazquez Rivera F., Martinez Lago N., Covela Rua M., Cousillas Castineiras A., Gonzalez Villarroel P., de la Camara Gomez J., Mendez Mendez J.C., Salgado Fernandez M., Candamio Folgar S. (2020). Efficacy and safety of trifluridine/tipiracil in third-line and beyond for the treatment of patients with metastatic colorectal cancer in routine clinical practice: Patterns of use and prognostic nomogram. Clin. Transl. Oncol..

[16] Carriles C., Jimenez-Fonseca P., Sánchez-Cánovas M., Pimentel P., Carmona-Bayonas A., García T., Carbajales-Álvarez M., Lozano-Blázquez A. (2019). Trifluridine/tipiracil (TAS-102) for refractory metastatic colorectal cancer in clinical practice: A feasible alternative for patients with good performance status. Clin. Transl. Oncol..

[17] Skuja E., Gerina-Berzina A., Hegmane A., Zvirbule Z., Vecvagare E., Purkalne G. (2018). Duration of previous treatment as a prognostic factor in metastatic colorectal cancer treated with trifluridine/tipiracil. Mol. Clin. Oncol..

[18] Kwakman J.J.M., Vink G., Vestjens J.H., Beerepoot L.V., De Groot J.W., Jansen R.L., Opdam F.L., Boot H., Creemers G.J., Van Rooijen J.M. (2018). Feasibility and effectiveness of trifluridine/tipiracil in metastatic colorectal cancer: Real-life data from The Netherlands. Int. J. Clin. Oncol..

[19] Cremolini C., Rossini D., Martinelli E., Pietrantonio F., Lonardi S., Noventa S., Tamburini E., Frassineti G.L., Mosconi S., Nichetti F. (2018). Trifluridine/tipiracil (TAS-102) in refractory metastatic colorectal cancer: A multicenter register in the frame of the Italian compassionate use program. Oncologist.

[20] Matsuda A., Yamada T., Matsumoto S., Sakurazawa N., Kawano Y., Shinozuka E., Sekiguchi K., Suzuki H., Yoshida H. (2019). Pretreatment neutrophil–to–lymphocyte ratio predicts survival after TAS-102 treatment of patients with metastatic colorectal cancer. Anticancer Res..

[21] Giuliani J., Bonetti A. (2019). The onset of grade ≥3 neutropenia is associated with longer overall survival in metastatic colorectal cancer patients treated with trifluridine/tipiracil. Anticancer Res..

[22] Eisenhauer E.A., Therasse P., Bogaerts J., Schwartz L., Sargent D., Ford R., Dancey J., Arbuck S., Gwyther S., Mooney M. (2009). New response evaluation criteria in solid tumours: Revised RECIST guideline (version 1.1). Eur. J. Cancer.

[23] National Cancer Institute Common Terminology Criteria for Adverse Events (CTCAE) v4.03. https://evs.nci.nih.gov/ftp1/CTCAE/CTCAE_4.03/CTCAE_4.03_2010-06-14_QuickReference_8.5x11.pdf.

[24] Panageas K.S., Ben-Porat L., Dickler M.N., Chapman P.B., Schrag D. (2007). When you look matters: The effect of assessment schedule on progression-free survival. J. Natl. Cancer Inst..

[25] Cicero G., De Luca R., Dieli F. (2018). Progression-free survival as a surrogate endpoint of overall survival in patients with metastatic colorectal cancer. OncoTargets Ther..

[26] Moriwaki T., Fukuoka S., Masuishi T., Takashima A., Kumekawa Y., Kajiwara T., Yamazaki K., Esaki T., Makiyama A., Denda T. (2020). Prognostic scores for evaluating the survival benefit of regorafenib or trifluridine/tipiracil in patients with metastatic colorectal cancer: An exploratory analysis of the REGOTAS study. Int. J. Clin. Oncol..

[27] Pietrantonio F., Miceli R., Rimassa L., Lonardi S., Aprile G., Mennitto A., Marmorino F., Bozzarelli S., Antonuzzo L., Tamburini E. (2017). Estimating 12-week death probability in patients with refractory metastatic colorectal cancer: The Colon Life nomogram. Ann. Oncol..

[28] Tanaka A., Sadahiro S., Suzuki T., Okada K., Saito G., Miyakita H. (2018). Retrospective study of regorafenib and trifluridine/tipiracil efficacy as a third-line or later chemotherapy regimen for refractory metastatic colorectal cancer. Oncol. Lett..

[29] Adenis A., De La Fouchardiere C., Paule B., Burtin P., Tougeron D., Wallet J., Dourthe L.-M., Etienne P.-L., Mineur L., Clisant S. (2016). Survival, safety, and prognostic factors for outcome with Regorafenib in patients with metastatic colorectal cancer refractory to standard therapies: Results from a multicenter study (REBECCA) nested within a compassionate use program. BMC Cancer.

[30] Chen D., Wu Y.-S., Lin H., Wang Y., Li L., Zhang T. (2018). Efficacy and safety of TAS-102 in refractory metastatic colorectal cancer: A meta-analysis. Cancer Manag. Res..

