# Supplementary material for: Early Clinical Experience with Trifluridine/Tipiracil for Refractory Metastatic Colorectal Cancer: The ROS Study
Source: Cancers (Basel). 2021 Sep 8;13(18):4514. doi: 10.3390/cancers13184514 (PMC8468101; doi:10.3390/cancers13184514)
Supplement: Supplementary file 1 [file cancers-13-04514-s001.zip › 2021_04_29_ROS_SuppTable2-Cancers_1.0.pdf]

Supplementary Table S2. Grade ≥3 treatment-related adverse events (*n*=379)

Adverse event	*n* (%)
Any grade ≥3 treatment-related adverse event	141 (37.2)
Grade ≥3 treatment-related adverse events[a]	
Neutropaenia (afebrile)	88 (23.2)
Anaemia	46 (12.1)
Thrombocytopaenia	20 (5.3)
Diarrhoea	15 (4.0)
Asthenia	13 (3.4)
Febrile neutropaenia	11 (2.9)
Nausea	8 (2.1)
Vomiting	5 (1.3)
Decreased appetite	3 (0.8)
Renal impairment	2 (0.5)

[a]Grade ≥3 treatment-related adverse events reported in >1 patient are presented.
