# Supplementary material for: Early Clinical Experience with Trifluridine/Tipiracil for Refractory Metastatic Colorectal Cancer: The ROS Study
Source: Cancers (Basel). 2021 Sep 8;13(18):4514. doi: 10.3390/cancers13184514 (PMC8468101; doi:10.3390/cancers13184514)
Supplement: Supplementary file 1 [file cancers-13-04514-s001.zip › 2021_08_02_ROS_SuppTable1-Cancers_1.0.pdf]

**Supplementary Table S1.** Trifluridine/tipiracil exposure and management ($n$=379)

| Treatment characteristics | Value |
|---|---|
| Planned dose at first cycle (mg/m$^2$, twice a day), median (IQR)[a] | 35.0 (35.0-35.0) |
| Treatment modification, *n* (%) | |
| Dose reduction | 116 (30.6) |
| Due to toxicity[b] | 108 (28.5) |
| Due to general state impairment | 6 (1.5) |
| Due to other reasons | 2 (0.5) |
| Dose delay | 191 (50.4) |
| Due to toxicity[c] | 167 (44.1) |
| Due to general state impairment | 11 (2.9) |
| Due to other reasons | 32 (8.4) |
| Reasons for end of treatment, *n* (%) | |
| Disease progression | 300 (79.2) |
| General state impairment | 48 (12.7) |
| Toxicity[d] | 17 (4.5) |
| Patient decision | 7 (1.8) |
| Other reasons | 7 (1.8) |
| Total number of administered cycles, median (IQR) | 3.0 (2.0-4.0)[e] |

*IQR* interquartile range.

[a]The planned dose at the first cycle was <35 mg/m$^2$ in 8 patients: 25 mg/m$^2$ $n$=1, and 30 mg/m$^2$ $n$=7.

[b]These 108 patients had 137 dose reductions due to the following toxicities: neutropaenia $n$=94, asthenia $n$=18, diarrhoea $n$=17, anaemia $n$=16, anaemia/thrombopaenia $n$=9, thrombopaenia $n$=5, bilirubin increase $n$=3, nausea/vomiting $n$=3, leucopaenia $n$=2, vomiting $n$=2, lymphocytopaenia $n$=1, mucositis $n$=1, nausea $n$=1, pancytopaenia $n$=1, and sickness/vomiting $n$=1 (multiple response variable, there may be more than one toxicity for each reduced dose).

[c]These 167 patients had 247 dose delays due to the following toxicities: neutropaenia $n$=197, anaemia $n$=25, diarrhoea $n$=13, asthenia $n$=12, thrombopaenia $n$=6, bilirubin increase $n$=5, leucopaenia $n$=2, nausea $n$=2, alkaline phosphatase $n$=1, alopecia $n$=1, anaemia/thrombopaenia $n$=1, bacterial infection $n$=1, fever $n$=1, liver

toxicity *n*=1, and vomiting *n*=1 (multiple response variable, there may be more than one toxicity for each delayed dose).

[d]These 17 patients reported end of treatment due to the following toxicities: neutropaenia *n*=6, anaemia *n*=3, diarrhoea *n*=2, asthenia *n*=2, anorexia *n*=1, atrial fibrillation *n*=1, cardiac toxicity *n*=1, impaired kidney function *n*=1, liver toxicity *n*=1, lumbalgia *n*=1, nausea *n*=1, pancytopaenia *n*=1, urinary tract infections favoured by neutropaenia *n*=1 (multiple response variable, there may be more than one toxicity for each ended treatment).

[e]Missing data *n*=1.
