# Supplementary material for: Early Clinical Experience with Trifluridine/Tipiracil for Refractory Metastatic Colorectal Cancer: The ROS Study
Source: Cancers (Basel). 2021 Sep 8;13(18):4514. doi: 10.3390/cancers13184514 (PMC8468101; doi:10.3390/cancers13184514)
Supplement: Supplementary file 1 [file cancers-13-04514-s001.zip › Fig supplementary.pdf]

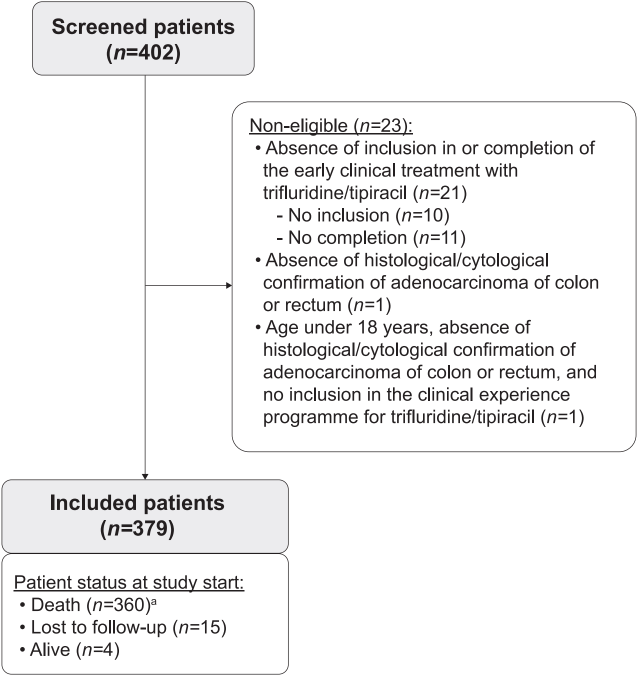

Supplementary Fig. 1 Summary of patient disposition. [a]Reasons for death included: progression (*n*=357), infection (*n*=1), pneumonia (*n*=1), and unknown (*n*=1).
